# Comparative Analysis of the GH/IGF-1 Axis during the First Sixth Months in Children with Low Birth Weight

**DOI:** 10.3390/children10121842

**Published:** 2023-11-24

**Authors:** Luciana Pessoa Maciel Diniz, Taisy Cinthia Ferro Cavalcante, Amanda Alves Marcelino da Silva

**Affiliations:** 1 Colegiado de Enfermagem Campus Petrolina, Universidade de Pernambuco, Petrolina 56328-900, PE, Brazil; luciana.diniz@upe.br; 2 Programa de Pós-graduação em Ciências da Saúde, Faculdade de Ciências Médicas, Universidade de Pernambuco, Recife 50100-130, PE, Brazil; taisy.cavalcante@upe.br

**Keywords:** infant, newborn, growth hormone, insulin-like growth factor 1, metabolic diseases, low weight newborn

## Abstract

Objective: To analyze the relation between alterations in the growth hormone (GH)/insulin-like growth factor 1 (IGF-1) axis during the first 6 months of life and weight in children born in the lower-middle São Francisco region. Methods: This is an analytical cohort and exploratory. Thirty children, were formed two groups, one of low birth weight children (LBW, *n* = 15) and another of normal weight (NBW = 15) were initially identified in a hospital and reapproached at 3 and 6 months of age. Birth weight and alterations in GH/IGF-1 curves were measured at birth and the third and sixth months of life. Results: Weight gain during the 6 months of follow-up in newborns with a low birth weight was greater compared to newborns with a normal birth weight. All children who were born with a low birth weight had an altered GH/IGF-1 curve at birth (*p* = 0.002). Most newborns with a low birth weight maintained the alteration in the GH/IGF-1 curve at the third month of life (*p* = 0.027). Regarding the GH/IGF-1 curve at the sixth month, alteration persisted in greater proportion among children with a low birth weight. Conclusions: Alterations in insulin resistance markers, demonstrated by increased GH without a proportional increase in IGF-1, were observed to be significant in children with a low birth weight with greater adiposity in this group which may increase the risk of metabolic diseases in later life.

## 1. Introduction

The World Health Organization considers low birth weight (LBW) to be one of the main causes of infant morbidity and mortality. LBW constitutes a serious public health problem, and it is one of the main causes of malnutrition, physical and developmental problems, as well as diabetes and cardiovascular diseases. In this context, clinical, pathological, and even social conditions may be linked to the condition of LBW, such as prematurity caused by intrauterine growth restriction, hypertensive diseases of pregnancy, hemorrhagic diseases, low-quality access to prenatal care, adolescence, and other conditions. The relationship of these conditions and variables can directly or indirectly contribute to significant conditions that may compromise a child’s health in the medium or long term, causing pathophysiological situations of risk to their health [1,2].

Studies have indicated that low weight can cause deficiencies in motor and intellectual development, emotional instabilities, difficulty developing pre-linguistic skills, and metabolic disorders such as obesity, diabetes, and cardiovascular diseases [1,2,3,4,5]. 

Studies have shown that most children between 5 and 10 years of age who were born with a LBW were already overweight/obese by preschool age; therefore, LBW and prematurity are an important risk factor for numerous health problems in childhood, adolescence, and adulthood [6,7].

Metabolic disorders, such as cardiovascular disease, diabetes, obesity, and others, may be related to genetic factors as well as to the fetal, prenatal, and postnatal environment [7]. This concept, called fetal programming, shows how the environment encountered before birth or during early stages of life can be related to and interfere with physiological processes, leading to endocrine disruption, for example, with consequent chronic alterations in later stages of life, such as metabolic syndrome [8,9].

Accordingly, a critical situation during the intrauterine period, such as a nutritional restriction leading to LBW, can result in alterations in the physiology of endocrine and metabolic processes that can have repercussions in adult life, such as obesity and other pathological conditions [10,11,12]. Thus, epigenetics and the interference of the environment in gene expression can explain the phenotypic plasticity of a fetus faced with nutritional restriction in order to survive, as well as the consequent predictive adaptive response [13,14].

The fetal period is the most influenced phase regarding the establishment of epigenetic variations, and, when facing environmental interference, these mechanisms can increase the expression of genes that deregulate hormones or markers, either increasing or decreasing them [15,16]. If a child is exposed to a hypercaloric postnatal diet without adequate nutrition or without the ingestion of breast milk, altered levels of hormones or biomarkers which have undergone epigenetic alterations when facing an unfavorable environment, such as leptin, growth hormone (GH) or insulin-like growth factor 1 (IGF-1), may favor metabolic actions outside appropriate physiological patterns, inducing obesity or other metabolic disorders in the medium and long term [14].

IGF-1 is a hormone that functions as the main mediator of somatic growth, stimulated by GH. It is also a mediator of GH-independent anabolic responses. Nutritional status is an important determinant of plasma IGF-1 [17,18]. GH, in turn, is produced by the somatotropic cells of the pituitary gland. GH production begins in early fetal life and continues throughout life, albeit at a progressively lower rate [19]. GH secretion is also affected by nutritional factors. It is increased in malnourished or fasting individuals, and it is stimulated by meals rich in proteins and amino acids administered intravenously [20].

GH is the main regulator of postnatal growth, and it has important metabolic actions. GH binds to its receptor, and, via activation of the JAK–STAT1 system, it stimulates the production of IGF-I, especially in the liver [19]. The role of GH in the regulation and its association with physiological and pathological conditions of metabolism has become increasingly consolidated. High levels of GH are related to increased lipolysis, increased insulin resistance and hyperglycemia, and increased levels of circulating free fatty acids, ketone bodies, and glucose, all of which are anabolic. It is, thus, in itself, an insulin resistance marker [19,20].

It is, therefore, understood that the integrated physiology of the GH/IGF-1 axis has regulatory somatic functions not only for growth, but also for the homeostasis of metabolic responses to external stimuli, especially nutritional ones. In addition to direct hereditary mechanisms, this regulation is sensitive to changes in expression resulting from the environment, mediated by epigenetic mechanisms [21]. According to this model, the GH/IGF-1 axis may undergo alterations induced by nutritional restriction, which affect the action of these hormones, causing complex metabolic alterations that may be associated with metabolic and cardiovascular diseases [14].

Up to the sixth month of life, nutritional behaviors and the type of breastfeeding can be considered important factors for a child’s nutritional status, which, added to the LBW condition, may increase risk factors in the management of future chronic conditions. In this context, elucidating this complex neuroendocrine mechanism triggered by LBW and its long-term consequences may, therefore, not only lead to direct scientific knowledge, but, above all, result in low-cost and highly effective public health measures. Therefore, we aimed to analyze the relationship between changes in the GH/IGF-1 axis during the first 6 months of life in children born in the lower-middle São Francisco Region in the northeast of Brazil.

## 2. Materials & Methods

Information collection took place following the signing of a free and informed consent form by the mother of the newborn, and the study received approval from the Ethics Committee of the Integrated Center Amaury de Medeiros (CISAM/UPE, acronym in Portuguese) under opinion number 4.728.276. This is an analytical cohort and exploratory. The children were initially identified at the Dom Malan Hospital, a regional reference institution in obstetrics and pediatrics, located in the municipality of Petrolina, Pernambuco, which is in the Sertão Region. The study included newborns from Petrolina, Pernambuco and the neighboring city of Juazeiro, Bahia.

The research participants were children with a LBW identified in the delivery room, surgery center, and during the rooming-in period of the Dom Malan Hospital and approached again at 3 and 6 months of age at their place of residence. The children identified or hospitalized in these sectors, even with a LBW, were in stable physiological conditions, which allowed a better approach and safe blood collection.

For this study, only the children’s weight was defined as a measurement for evaluation. This sole measurement is adopted by the Brazilian Ministry of Health to assess underweight, overweight, and obesity. Thus, children weighing less than 2500 g were considered LBW [22].

Blood samples were collected by peripheral vein puncture, and laboratory analyses were carried out in a reference laboratory using the chemiluminescence technique. GH was evaluated without stimulation and used as a parameter for comparison between birth and the third and sixth months of life.

As this was a cohort that sought to compare groups exposed and not exposed to the condition of LBW and, based on this, investigate the outcome of presence of insulin resistance markers, for each child with LBW, a child with adequate weight was approached.

Initially, at month zero, 30 newborns were approached. After the third and sixth months, there was the loss of 5 children, due to parents giving up, death, or loss of contact. Weight gain variables were created for the following 3 moments: weight gain during the first 3 months of the newborn’s life (calculated by subtracting birth weight from weight at the third month); weight gain from the third to the sixth month of the newborn’s life (calculated by subtracting weight at the third month from weight at the sixth month), and weight gain during the first 6 months of the newborn’s life (calculated by subtracting birth weight from weight at the sixth month). These 3 new variables were treated numerically. The other variables used (numerical and categorical) were treated in their original form as arranged in the database.

Initially, univariate descriptive analysis of the variables was conducted according to their classification. For categorical variables, frequency distribution in absolute and relative numbers was used. For numerical variables, measures of central tendency (mean) and dispersion (minimum, maximum, and standard deviation [SD]) were presented. The normality of the distribution of numerical variables was verified using the Shapiro–Wilk test, which is appropriate for small samples.

Normality tests are widely used in statistical procedures to help the user choose the type of test to be used or to validate some assumption required by the technique [23]. There are two ways to test normality, the first through graphical methods, the second through numerical methods, such as the application of specific statistical tests [23]. Thus, for variables with normal distribution, parametric tests were applied. For variables that did not show normal distribution (*p* < 0.05), non-parametric variables were applied in inferential analysis. 

In the bivariate analyses involving numerical variables that showed normal distribution, Student’s *t*-test was used. For crossings that did not meet the parametric assumptions, the Mann–Whitney test, which is the non-parametric equivalent of Student’s *t*-test, was used. Correlations between the variables were verified using Spearman’s and Pearson’s correlation tests, verifying the rho and r coefficient signs, respectively, and statistical significance using the *p* value [24]. For statistically significant correlations, scatter plots were constructed for better visualization of the relationship between the analyzed variables.

The associations between the study variables were tested using Pearson’s chi-square and/or Fisher’s exact test, which is indicated when one intends to test the hypothesis that frequency data are distributed according to some theory or postulate. However, some criteria must be considered, including the sample size and the expected frequency values [25]. The test was defined considering the expected frequency obtained with the crossing, in which, for expected frequencies less than 5, Fisher’s exact test was used in 2 × 2 contingency tables. When the expected frequency was less than 5 in up to 20% of cells, 2 × 3 or greater contingency tables were accepted [24].

If the assumptions for using the chi-square test were not met, Fisher’s exact test was adopted. For all tests, significance level of 5% and confidence interval of 95% were adopted. Stata 14.0 statistical software and Microsoft Office Excel 365 (https://www.office.com) were used to generate the tables.

## 3. Results

Analyzing the characteristics of gestational age, weight, and weight gain of the newborns at birth and the measurements at the third and sixth months, it was possible to observe that the mean gestational age was 37 weeks; the mean birth weight was 2616 g (SD = 830 g); at the third month, the mean weight was 6744 g (SD = 757 g); and, at the sixth month, the mean weight was 9298 g (SD = 1420 g). Mean weight gain during the period between birth and the sixth month was 6670 g (SD = 1627 g) for all newborns. The mean length of hospital stay was 3 days (SD = 7).

When comparing the characteristics of newborns between those with normal birth weight and LBW, a significant difference was observed in gestational age; those with a LBW were preterm (gestational age 35 weeks; SD = 3), and those with normal birth weight went to term (gestational age 39 weeks; SD = 1). Weight measurements of newborns at the third and sixth months were not statistically different (*p* > 0.05). However, the weight gain at 6 months of follow-up among newborns with LBW was greater (mean weight gain of 7559 g; SD = 1443 g), compared to newborns with normal birth weight (mean weight gain of 5708 g) (*p* = 0.004) (Table 1).

When analyzing birth and clinical characteristics, it was observed that all newborns who were born with LBW were preterm (*p* = 0.000) and all newborns with a normal weight and the majority of newborns with a LBW were born by vaginal delivery (*p* = 0.017). None of the newborns with normal birth weight and the majority of newborns with a LBW had complications (*p* = 0.006). All newborns with a LBW had an altered GH/IGF-1 curve at birth (*p* = 0.002). The majority of newborns with a LBW maintained the alteration in the GH/IGF-1 curve at the third month of life (*p* = 0.027). Regarding the GH/IGF-1 curve at the sixth month of life, the majority remained normal, although a high proportion still maintained the altered curve at the sixth month (42.9%; *p* = 0.017). Of the newborns with a LBW, only 1 (6.7%) breastfed within the first hour of life (*p* = 0.000), the main reason being respiratory distress among the newborns with a LBW (*p* = 0.010) (Table 2).

The results of GH levels at birth showed a significant difference; newborns with a LBW had a higher mean value (GH = 19.4; SD = 7.9) when compared to newborns with a normal birth weight (GH = 11.9; SD = 9.4) (*p* = 0.011). The same scenario was observed in the GH values at the sixth month. Newborns with a LBW had a higher average (GH = 5.3; SD = 4.8) compared to those with a normal birth weight (GH = 1.3; SD = 1.1) (*p* = 0.008). The other results did not show significant differences in their values (Table 3). 

Considering the monitoring of all newborns’ weight gain over the first 6 months of life, a positive correlation was observed with GH at the 6th month (rho 0.58; *p* = 0.002). For newborns with a LBW, not only was there a positive correlation between weight gain during the first 6 months; there was also an increase in the strength of the correlation with GH at the 3rd and 6th months (rho = 0.49; *p* = 0.030; and rho = 0.69; *p* = 0.009;) (Table 4).

**Figure 1:**
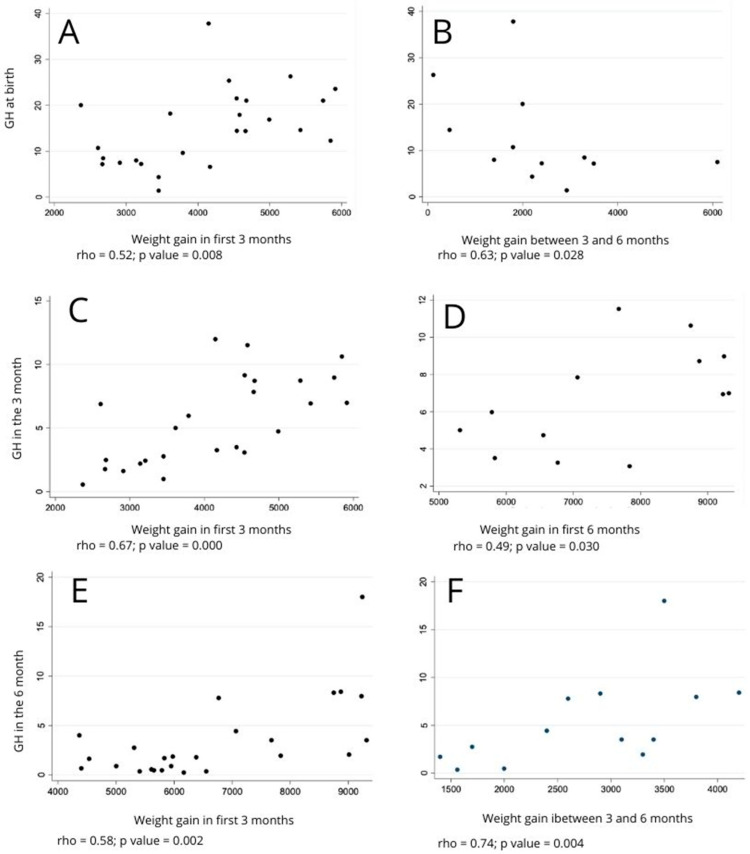
Correlations between GH level and weight gain. (**A**): GH at birth and weight gain from 0 to 3 months in all newborns; (**B**): GH at birth and weight gain from 3 to 6 months in newborns with normal birth weight; (**C**): GH at 3 months and weight gain from 0 to 3 months in all newborns; (**D**): GH at 3 months and weight gain from 0 to 6 months in newborns with low birth weight; (**E**): GH at 6 months and weight gain from 0 to 6 months in newborns with low birth weight; (**F**): GH at 6 months and weight gain from 3 to 6 months in newborns with low birth weight. GH: growth hormone.

Verifying the correlation between the weight gain of newborns and the values obtained in GH and IGF-1, the greater the weight gain during the first 3 months for all newborns, the greater the GH at birth (rho = 0.52; *p* = 0.008; Figure 1A) and GH at the third month of life (r = 0.67; *p* = 0.000; Figure 1C). For newborns with a normal birth weight, GH at the third month also increased as the child’s weight (r = 0.72; *p* = 0.009; Figure 1D), while the IGF-1 value decreased as weight gain took place during the first 3 months of life (r = −0.63; *p* = 0.029).

The weight gain observed between the third and sixth month was positively correlated with the level of IGF-1 at the third month (r = 0.47; *p* = 0.018) and GH at the sixth month (rho = 0.53; *p* = 0.007). For newborns with a LBW, the greater the weight gain between the 3rd and 6th month of life was, the greater the GH values collected at the sixth month were (rho = 0.74; *p* = 0.004; Figure 1E,F). For newborns with a normal weight, the weight gain in this interval between the third and sixth month decreased as the amount of GH was higher at birth (rho = −0.63; *p* = 0.028; Figure 1B).

Figure 1 displays comparison of GH levels at months 0, 3, and 6, as well as its relations to weight gain in both populations.

## 4. Discussion

Data analysis made it possible to corroborate the perspective that a LBW exerts a direct influence on altered GH levels, which are maintained during the first 6 months of life. The increase in GH levels, without an accompanying increase in the levels of IGF-1, leads to an imbalance of the GH/IGF-1 axis, as it is an insulin resistance marker. This can cause changes in adiposity, which may increase the risk of metabolic disorders, in future phases, in children with a LBW.

In this study, it was possible to verify laboratory alterations in the analyzed markers as early as the month of birth, with prevalence in newborns with a LBW. These alterations are in agreement with studies that have demonstrated that metabolic disorders or increased risks for obesity, diabetes, and cardiovascular diseases identified in adolescence or in adult life may be associated with or be diagnosed at increasingly earlier stages of life, mediated by clinical evaluations and laboratory tests in underweight or premature patients [26]. It is believed that the metabolic system peaks in its developmental process during the perinatal period and that critical or unfavorable conditions during this phase, such as those that lead to restrictive growth that leads to LBW, can reprogram the endocrine system, with repercussions throughout life [27,28,29].

Conditions that reflect nutrition below optimal levels, causing LBW, can lead to changes in gene expression, even in the intrauterine environment or during early postnatal stages [30,31,32]. Accordingly, the hypothesis of fetal programming gains strength and robustness when analyzing which environmental aspects can impact genetic regulation, thus interfering with hormonal markers that induce the accumulation and distribution of adipose tissue in adult life [33,34].

In this context, the periods considered stressful or critical for fetal development would lead to a pre-programming that induces concentrations outside the normal hormone, neurotransmitter, and metabolic curves, leading to metabolic changes that will be reflected throughout life; an example of this occurs when facing LBW, generating changes in insulin markers that can be identified early [35].

The so-called adaptations between a critical environment and the need for physiological development are related to hormonal changes or biochemical biomarkers that, in this environment of nutritional inadequacy, would be necessary for the survival of the fetus [36]. However, this genetic plasticity, which is necessary for survival adaptation, can, in the long term, be converted into altered metabolism and, consequently, lead to the emergence of metabolic conditions and deficiencies, such as diabetes and obesity. Accordingly, LBW and prematurity have shown a significant relationship with increased risk of developing non-communicable chronic diseases [26].

It is worth highlighting that the association between LBW and overweight in children who are born prematurely, while examining the effect of adjustment for body measurements, can lead to spurious results regarding the association when sought during the child’s development [37]. This study, however, directly investigated the association between LBW and the GH/IGF-1 curve.

The research found that the changes in GH, with increased levels, were significant among newborns with a LBW from birth to the sixth month, which reflects the altered axis within this population, in addition to a more pronounced weight gain when compared with children with an adequate birth weight. This change is evident in children with a LBW, whose increased GH levels were not associated with a proportional increase in IGF-1, leading to an alteration of the GH/IGF-1 axis [14]. This phenomenon, justified by epigenetic alterations with the aim of increasing fat concentration and maintaining survival, can persist for 3 months or more after birth in the absence of adequate nutritional supply. It is believed that this altered endocrine state leads to increased visceral adiposity, changes in lipid levels, and the presence of other components of metabolic syndrome at later ages [38].

A retrospective study carried out in the 1980s in the United Kingdom demonstrated a correlation between metabolic disorders, such as cardiovascular diseases, and increased mortality in children born weighing less than 2500 g. These studies have shown that a LBW can lead to an altered distribution of physiological patterns and culminate in hormonal, blood, and pancreatic alterations through a process of intrauterine adaptation with the aim of fetal survival in an environment with low nutritional resources [39].

This process, with the initial objective of adaptation, can cause gene-mediated reprogramming that would lead to alterations in insulin sensitivity, defects in the secretion or response of markers, or an association of both factors that would generate diseases at later times. This phenomenon has been found in children who are small for gestational age and in those who are premature or who have LBW [40,41].

Thus, conditions with low nutrient availability or under fasting, for example, GH secretion levels would be increased, which would configure a predominance of GH levels in low weight situations, for example. In this situation, the levels of IGF-I and insulin concentrations are low and those of free fatty acids are elevated [42]. In situations of LBW, for example, with scarce carbohydrate storage, lipolysis, which increased due to high GH levels, becomes necessary to obtain energy to save protein and ensure survival during periods of food scarcity. This metabolic stress induces alterations in the GH and IGF-1 levels and, with this, lipid, endocrine, and anabolic consequences in order to maintain the physiological conditions for survival. This increased cascade of alterations, however, leads to damage in gene expression that can be translated into important metabolic changes in the future [43].

It is known that body adiposity during postnatal periods has been associated with children with intrauterine growth retardation, with a consequent LBW [44]. Accordingly, there is evidence that children with lower birth weight were 2.5 times more likely to have metabolic syndrome in adulthood, increasing the risk of metabolic syndrome at older ages. This fact is related to previously described alterations in the GH/IGF-1 axis that increase fat breakdown, due to a disproportionate increase in GH in relation to IGF-1, leading to fat accumulation and insulin resistance, which are the main mechanisms suggested for metabolic syndrome [45].

There is a correlation showing that environmental factors condition important changes for the development of obesity and all the metabolic risks that this entails. These changes are related to genetic mechanisms resulting from and caused by stressful situations, even in the intrauterine environment [44]. These genetic factors contribute to the relationship between birth weight and chronic diseases in adults by permitting alterations in genetic patterns that ensure adaptation for survival in a possibly inhospitable environment for the fetus, which could lead to death [46]. With modifications in adipose tissue, through the activation of biochemical pathways, there is a change in the role of regulating energy stores, favoring insulin resistance in a favorable postnatal environment, leading to feedback that favors greater adiposity [36].

Further evidence in this sense involves the so-called sparing phenotype or predictive adaptive response, indicating that individuals exposed to reduced intrauterine nutritional supply, who respond with reduced growth as reflected by their LBW, show alterations in epigenetic mechanisms during the prenatal phase [47]. These mechanisms may be associated with methylation, which induces increased GH levels, which would lead to increased insulin resistance, increasing cell adiposity and, thus, providing better survival conditions in a restrictive environment. In later stages of life, this epigenetic alteration that occurred in utero and led to LBW may still be present, which would considerably increase obesity, diabetes, or cardiovascular disorders [48,49].

It is, however, known that the rapid weight gain observed after the birth of children with LBW, after unfavorable conditions suffered in utero, guarantees better recovery for their neural and immunobiological development [50]. However, this additional increase in weight gain, which is even more evident when it is not the result of adequate nutrition, such as exclusive breastfeeding, further contributes to the accumulation of fat and increased insulin resistance, due to the epigenetic mechanisms undergone while still in the pre-life phase. Thus, this further contributes to the increased risk of developing cardiometabolic diseases throughout life [51].

Considering this scenario, monitoring the physiological evolution of fetal development, in addition to acquiring clinical and laboratory data at birth and during the first postnatal months can greatly contribute to the risk analysis of a child’s future health conditions [14]. Identifying mechanisms that can interfere with epigenetics can assist in the identification and analysis of risk factors for the development of future chronic diseases. The importance of the attention given to the intrauterine phase is due to the fact that these mechanisms, according to studies conducted in recent years, may be related to events that occurred in utero, as a response to an environmental stressor event, an example of which would be the low supply of nutrients that resulted in LBW [52].

Thus, it was possible in this study to observe the existence of a relationship between LBW and alterations in the curve of the GH/IGF-1 axis. It is feasible that there was greater weight gain among children born weighing less than 2500 g compared to children with a normal birth weight. There was a significant positive relationship between greater weight gain in that group and the GH levels above reference values. 

Accordingly, it was observed that altered insulin resistance markers, demonstrated in this study by the increase in GH without a proportional increase in IGF-1, may induce epigenetic alterations that are capable of increasing the risk of the installation of metabolic diseases in subsequent stages of life. 

The results of this research, in addition to the multiple studies cited, demonstrated a likely relationship between LBW and changes in the GH/IGF-1 axis, which makes it possible to speculate that this condition at birth may be associated with the risk of developing future diseases, especially endocrine diseases. Nevertheless, further in-depth population studies that address this subject and consider the variables involved in future conditions of life are fundamental to the causal confirmation of this hypothesis. 

This becomes increasingly pertinent, because, when identifying risk factors that may lead to future chronic diseases caused during earlier stages of life, health promotion and prevention actions can be carried out in children with LBWs with the aim of reducing morbidity and mortality, thus improving this population’s quality of life.

## Figures and Tables

**Table 1:** Birth characteristics of newborns. Petrolina, Pernambuco, Brazil, 2021.

	All Newborns					Newborns with Normal Birth Weight					Newborns with Low Birth Weight					*p* Value
	*n*	Mean	Standard Deviation	Minimum	Maximum	*n*	Mean	Standard Deviation	Minimum	Maximum	*n*	Mean	Standard Deviation	Minimum	Maximum	
Approximate gestational age	30	37	3	27	41	15	39	1	37	41	15	35	3	27	36	0.000 *
Birth weight	30	2616	830	965	3985	15	3324	466	2520	3985	15	1908	368	965	2465	0.000 **
Weight at 3 months	25	6744	757	5400	8000	12	6704	805	5700	8000	13	6780	740	5400	7900	0.808 **
Weight at 6 months	25	9298	1420	7100	13,000	12	9038	1447	7495	13,000	13	9538	1407	7100	11,300	0.390 **
Weight gain at 3 months	25	4116	1080	2370	5915	12	3374	880	2370	5290	13	4800	752	3615	5915	0.000 **
Weight gain from the third to the sixth month	25	2555	1252	120	6100	12	2334	1562	120	6100	13	2758	898	1400	4200	0.408 **
Total weight gain (from birth to the sixth month)	25	6670	1627	4370	9315	12	5708	1250	4370	9015	13	7559	1443	5315	9315	0.004 *
Length of hospital stay, days	30	3	7	0	22	15	1	1	0	1	15	5	9	0	22	0.622 *

* Mann–Whitney test; ** Student’s *t*-test.

**Table 2:** Sociodemographic and clinical characteristics of newborns. Petrolina, Pernambuco, Brazil, 2022.

	All Newborns		Newborns with Normal Birth Weight		Newborns with Low Birth Weight		*p* Value
	*n*	%	*n*	%	*n*	%	
Gestational age classification							
Preterm (<37 weeks)	16	53.3	1	6.7	15	100.0	0.000 *
Term (37 to 41 weeks)	14	46.7	14	93.3	-		
Sex							
Male	15	50.0	7	46.7	8	53.3	0.715 *
Female	15	50.0	8	53.3	7	46.7	
Classification of weight at 3 months							
Normal weight	18	75.0	8	72.7	10	76.9	1.000 **
Overweight	6	25.0	3	27.3	3	23.1	
Classification of weight at 6 months							
Normal weight	14	56.0	8	66.7	6	46.2	0.233 **
Overweight	5	20.0	3	25.0	2	15.4	
Obesity	6	24.0	1	8.3	5	38.5	
Classification of birth weight							
Normal weight	15	50.0	15	100.0	-		0.000 *
Low weight	15	50.0	-		15	100.0	
Type of delivery							
Vaginal	24	80.0	15	100.0	9	60.0	0.017 **
Cesarean	6	20.0	-		6	40.0	
Complications during delivery							
No	23	76.7	15	100.0	8	53.3	0.006 **
Yes	7	23.3	-		7	46.7	
GH/IGF-1 curve, at birth							
Normal	7	28.0	7	58.3	-		0.002 **
Altered	18	72.0	5	41.7	13	100.0	
GH/IGF-1 curve, at 3 months							
Normal	13	52.0	9	75.0	4	30.8	0.027 *
Altered	12	48.0	3	25.0	9	69.2	
GH/IGF-1 curve, at 6 months							
Normal	20	76.9	12	100.0	8	57.1	0.017 **
Altered	6	23.1	-		6	42.9	
Postpartum referral							
Rooming-in	20	66.7	15	100.0	5	33.3	0.000 **
Internal nursery	8	26.7			8	53.3	
Intensive care unit	2	6.7			2	13.3	
Breastfed within the first hour of life							
No	17	56.7	3	20.0	14	93.3	0.000 *
Yes	13	43.3	12	80.0	1	6.7	
Reason for not breastfeeding with the first hour							
Mother not producing milk	1	5.9	1	33.3			0.010 **
Respiratory distress	10	58.8			10	71.4	
Mother referred to the intensive care unit and respiratory distress	2	11.8			2	14.3	
Newborn dyspnea	1	5.9			1	7.1	
Lethargic newborn	2	11.8	2	66.7			
Ventilatory support	1	5.9			1	7.1	

* Pearson’s chi-squared test; ** Fisher’s exact test.

**Table 3:** Analysis of GH and IGF-1 results for all newborns and association between newborns with normal weight and low birth weight.

	All Newborns					Normal Weight					Low Weight					*p* Value
	*n*	Mean	SD	Min	Max	*n*	Mean	SD	Min	Max	*n*	Mean	SD	Min	Max	
GH ^&^ result, at birth	30	15.6	9.4	1.4	37.8	15	11.9	9.4	1.4	37.8	15	19.4	7.9	6.6	34.8	0.011 *
IGF-1 ^&^ result, at birth	30	43.0	19.0	14.0	78.0	15	44.9	20.8	18.0	78.0	15	41.1	17.6	14.0	76.0	0.593 **
GH ^&^ result, third month	25	5.5	3.5	0.6	12.0	12	4.3	3.8	0.6	12.0	13	6.7	2.8	3.1	11.5	0.083 **
IGF-1 ^&^ result, third month	25	50.9	19.8	20.0	91.0	12	45.9	18.9	20.0	71.0	13	55.5	20.2	28.0	91.0	0.236 **
GH ^&^ result, sixth month	25	3.4	4.0	0.2	18.0	12	1.3	1.1	0.2	4.0	13	5.3	4.8	0.3	18.0	0.008 *
IGF-1 ^&^ result, sixth month	25	44.0	28.9	15.0	126.0	12	44.8	28.6	20.0	126.0	13	43.2	30.3	15.0	97.0	0.479 *

* Mann–Whitney test; ** Student’s *t*-test; ^&^ values shown in ng/mL.

**Table 4:** Correlation between GH and IGF-1 values at birth, 3 months, and 6 months and weight gain of newborns according to weight classification.

	GH ^&^ at Birth		IGF-1 ^&^ at Birth		GH ^&^ at 3 Months		IGF-1 ^&^ at 3 Months		GH ^&^ at 6 Months		IGF-1 ^&^ at 6 Months	
	Coef.	*p* Value	Coef.	*p* Value	Coef.	*p* Value	Coef.	*p* Value	Coef.	*p* Value	Coef.	*p* Value
**All newborns**												
Weight gain during the first 3 months	0.52	0.008 **	−0.21	0.323 *	0.67	0.000 *	0.09	0.686 *				
Weight gain between the third and sixth month	−0.16	0.431 **	0.10	0.627 *	−0.15	0.472 *	0.47	0.018 *	0.53	0.007 **	−0.02	0.913 **
Weight gain during the first 6 months	0.14	0.514 **	−0.06	0.782 *	0.29	0.154 **	0.39	0.054 **	0.58	0.002 **	−0.07	0.732 **
**Newborns with low birth weight**												
Weight gain during the first 3 months	0.16	0.591 **	−0.01	0.972 *	0.48	0.099 *	0.40	0.171 *				
Weight gain between the third and sixth month	0.17	0.578 **	−0.02	0.946 *	0.48	0.093 *	0.34	0.253 *	0.74	0.004 **	0.09	0.760 **
Weight gain during the first 6 months	0.27	0.364 **	−0.02	0.952 *	0.49	0.030 **	0.39	0.187 **	0.69	0.009 **	0.10	0.753 **
**Newborns with normal birth weight**												
Weight gain during the first 3 months	0.15	0.640 **	−0.45	0.144 *	0.72	0.009 *	−0.63	0.029 *				
Weight gain between the third and sixth month	−0.63	0.028 **	0.18	0.586 *	−0.56	0.060 *	0.55	0.061 *	0.22	0.485 **	−0.27	0.397 **
Weight gain during the first 6 months	−0.52	0.085 **	−0.10	0.766 *	−0.04	0.897 *	0.17	0.587 **	0.01	0.983 **	−0.31	0.330 **

* Pearson’s correlation; ** Spearman’s correlation; ^&^ values shown in ng/mL.; Coef. Correlation coefficient, namely, rho for Spearman’s correlation and r for Pearson’s correlation.

## Data Availability

The data presented in this study are available on request from the corresponding author. The data are not publicly available due to privacy.
